# Supplementary material for: Engineering of phenylalanine dehydrogenase from Thermoactinomyces intermedius for the production of a novel homoglutamate
Source: PLoS One. 2022 Mar 30;17(3):e0263784. doi: 10.1371/journal.pone.0263784 (PMC8967036; doi:10.1371/journal.pone.0263784)
Supplement: S1 Fig — (a) Effect of PMSF on substrate decreased the residual enzyme activity. (b) Effect of different concentration of Trehalose and Sucrose showed protective effects in the stability of the enzyme. (DOCX) [file pone.0263784.s001.docx]

**
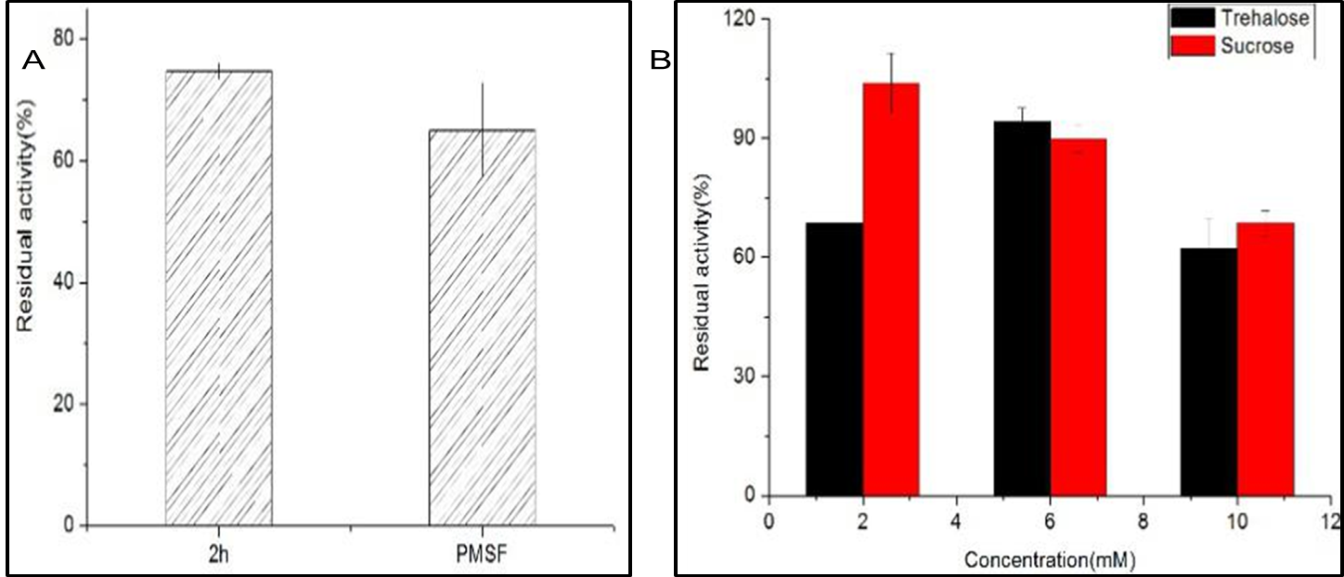
**

**S1 Fig.** (**a**) Effect of PMSF on substrate decreased the residual enzyme activity (**b**) Effect of different concentration of Trehalose and Sucrose showed protective effects in the stability of the enzyme.
