# Supplementary material for: Engineering of phenylalanine dehydrogenase from Thermoactinomyces intermedius for the production of a novel homoglutamate
Source: PLoS One. 2022 Mar 30;17(3):e0263784. doi: 10.1371/journal.pone.0263784 (PMC8967036; doi:10.1371/journal.pone.0263784)
Supplement: S2 Fig — The red underlined K69, K81, and N264 are conserved residues and are involved in the binding of substrate. Whereas the highlighted residues encircled in the red boxes are involved in the binding of cofactors. (DOCX) [file pone.0263784.s002.docx]

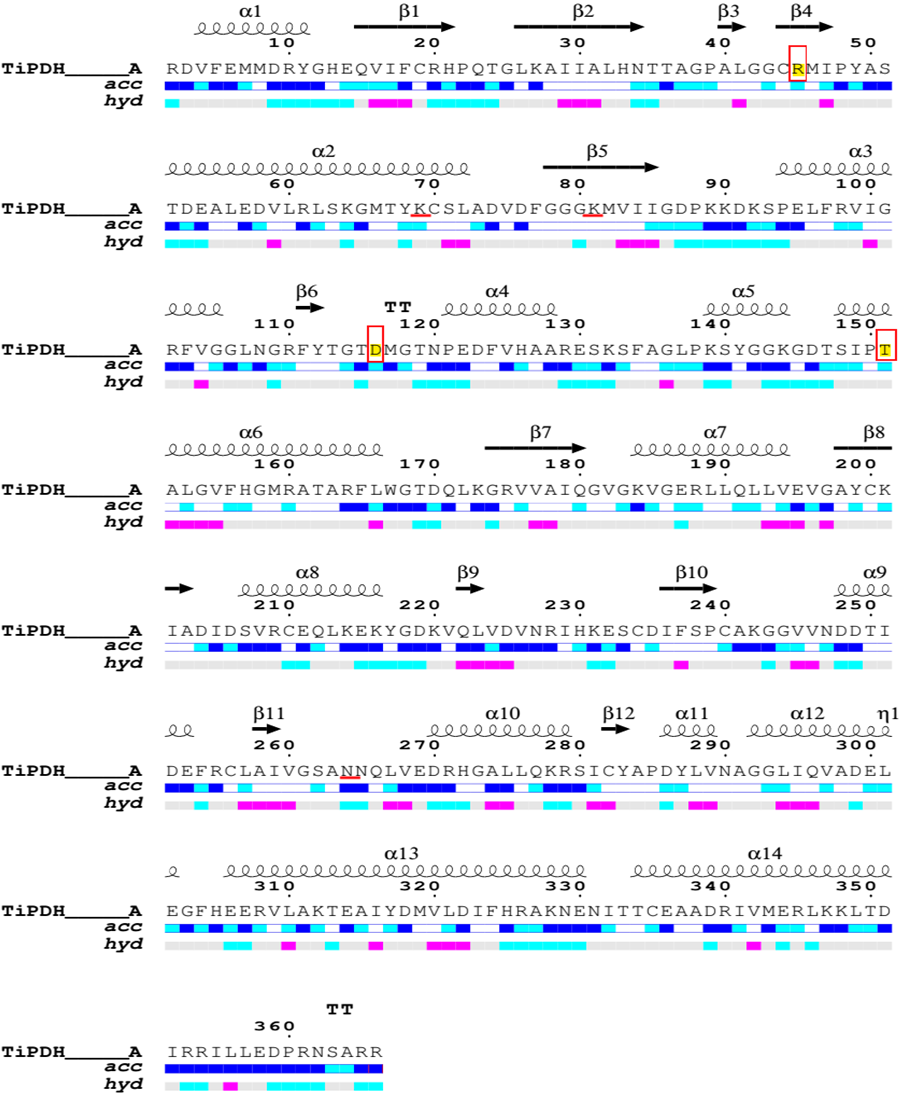


**S2 Fig. TiPDH sequence containing thirteen α helices and twelve β strands sheets and some conserved regions.** The red underlined K69, K81, and N264 are conserved residues and are involved in the binding of substrate. Whereas the highlighted residues encircled in the red boxes are involved in the binding of cofactors.
