# Supplementary material for: Engineering of phenylalanine dehydrogenase from Thermoactinomyces intermedius for the production of a novel homoglutamate
Source: PLoS One. 2022 Mar 30;17(3):e0263784. doi: 10.1371/journal.pone.0263784 (PMC8967036; doi:10.1371/journal.pone.0263784)
Supplement: S1 Table — (DOCX) [file pone.0263784.s003.docx]

**S1 Table. Plasmids used in this work.**

**Plasmids Descriptions**

pETduet-1-TiLDH pETDuet-1, leucine dehydrogenase gene from *Thermoactinomyces intermedius*

pETduet-1-SuPDH pETDuet-1, phenylalanine dehydrogenase gene from *Sporosarcina ureae*

pETduet-1-TiPDH pETDuet-1, phenylalanine dehydrogenase gene from *Thermoactinomyces intermedius*

pETduet-1-BsGDH pETDuet-1, glutamate dehydrogenase gene from *Bacillus subtilis*

pETduet-1-DAPDH pETDuet-1, *meso*-diaminopimelate dehydrogenase gene from *Symbiobacterium thermophilum*

pETduet-1-Fdh pETDuet-1,formate dehydrogenase gene from *Candida boidinii*
