# Supplementary material for: Engineering of phenylalanine dehydrogenase from Thermoactinomyces intermedius for the production of a novel homoglutamate
Source: PLoS One. 2022 Mar 30;17(3):e0263784. doi: 10.1371/journal.pone.0263784 (PMC8967036; doi:10.1371/journal.pone.0263784)
Supplement: S2 Table — (DOCX) [file pone.0263784.s004.docx]

**S2 Table. Primers for wild type and mutant enzymes used in this study.**

**Primers Sequence 5'-3'**

*pETduet-1-TiLDH-F* GGGAAAG*GATCCG*ATGAAAATCTTCGATTACATGGA

*pETduet-1-TiLDH-R* GGGAAA*CTCGAG*TTATTTGTTGTTAAAATTGATCAGGT

*pETduet-1-TiPDH-F* GGGAAAG*AATTCG*ATGCGCGACGTGTTTGAAATGATG

*pETduet-1-TiPDH-R* GGGAAA*CTCGAG*TTACCTCCTTGCGCTGTTGCG

*pETduet-1-SuPDH-F* GGGAAAG*GATCCG*ATGATTTTGGTAACTTTAGAACAGACT

*pETduet-1-SuPDH-R* GGGAAA*CTCGAG*TTACTGATGAAAATTCCATTTCGGTC

*pETduet-1-BsGDH-F* GGGAAAG*GATCCG*ATGTCAGCAAAGCAAGTCTCGAAAG

*pETduet-1-BsGDH-R* GGGAAA*CTCGAG*TTAGACCCATCCGCGGAAACGC

*pETduet-1-DAPDH-F* GGGAAA *GGATCC*GGATAAACTGCGTGTTG

*pETduet-1-DAPDH-R* GGGAAA *GGTACC*TTAAACCAGTTTACGAATCCAT

*pETduet-1-Fdh-F* GGGAAA*GGATCC*GAAAATTGTGCTGGTGTTATATGAT

*pETduet-1-Fdh-R* GGGAAA*GGTACC*TCACTTTTTATCATGTTTTCCGTAC

*TiPDH-A135R-F* CCAGGGAATCGAAATCTTTTcgCGGATTGCCGAAATCGTACG

*TiPDH-A135R-R* CGTACGATTTCGGCAATCCGcgAAAAGATTTCGATTCCCTGG

*TiPDH-G114R-F* TAAACGGCCGTTTCTATACCcGcACCGACATGGGAACCAATCC

*TiPDH-G114R-R* GGATTGGTTCCCATGTCGGTgCgGGTATAGAAACGGCCGTTTA
