# Supplementary material for: Engineering of phenylalanine dehydrogenase from Thermoactinomyces intermedius for the production of a novel homoglutamate
Source: PLoS One. 2022 Mar 30;17(3):e0263784. doi: 10.1371/journal.pone.0263784 (PMC8967036; doi:10.1371/journal.pone.0263784)
Supplement: S3 Table — (DOCX) [file pone.0263784.s005.docx]

**S3 Table. Molecular docking statistics of α-Ketoadipic acid and Phenylalanine with the binding pocket of TiPDH.**

| **Compound and protein** | **Hydrogen bonding residues and distance** | **Hydrophobic Interacting residues** | **B. Affinity (Kcal/mol)** |
| --- | --- | --- | --- |
| α-Ketoadipic acid and TiPDH | Gly145 (2.80 Å), Thr147 (2.92 Å) and Gly293 (2.79 Å) | Gly114, Thr115, Ala135, Gly136, Lys144, Asp146, Gln296, Val297 and Glu300 | -4.8 |
| Phenylalanine and TiPDH | Asn264 (2.97 Å), Thr115 (2.97 Å) | Leu41, Gly42, Gly43, Met66, Lys69, Gly114 and Leu294 | -3.5 |
