# Supplementary material for: Engineering of phenylalanine dehydrogenase from Thermoactinomyces intermedius for the production of a novel homoglutamate
Source: PLoS One. 2022 Mar 30;17(3):e0263784. doi: 10.1371/journal.pone.0263784 (PMC8967036; doi:10.1371/journal.pone.0263784)
Supplement: Attachment — Submitted filename: Evaluation AAA.docx [file pone.0263784.s006.docx]

**General Comments**

The work “Engineering of phenylalanine dehydrogenase…” PONE D 21 21697, by Tariq et al. is dedicated to the design of an enzymatic system to obtain the non-proteinogenic amino acid homoglutamate. This compound may have medical interest and is usually known as alpha aminoadipic acid (AAA). In the Introduction the authors describe several AAA biosynthetic methods that may be used to obtain this compound; however, they do not focus the article on the use of the naturally producing AAA biosynthetic enzymes, the names of which are not cited. These enzymes are present in actinobacteria or fungi and are well known to form AAA as precursor of the beta-lactam antibiotics. Instead, the authors choose to study the phenylalanine dehydrogenase of *Thermoactinomyces intermedius* an enzyme that, as the authors indicate in line 336, lacks efficiency for the amination of alpha cetoadipic acid, the compound used by the authors as substrate. Therefore, it is surprising that the authors choose to modify the phenylalanine DH which belong to type I (aromatic) aminotransferases to develop this project. The experimental part of the work dedicated to the modification of the *T intermedius* PDH is largely correct although there are some obscure points in the quantification method as indicated below. The authors also studied the regeneration of NADH cofactor for the TiPDH by using the well known formate DH (decarboxylase) that produces NADH. The discussion is entirely focused on the modification of the protein that the authors have performed but they fail to discuss the main aim of this project which is the microbial production of AAA by microbial biosynthetic methods which are easy and highly productive.

**Specific comments**

1. In the abstract the authors refer to the compound of interest as homoglutamate; however, this compound is usually named 2-aminoadipic acid or alpha aminoadipic acid. Please, use at least in the abstract the two names, so that the readers can identify the compound that is being investigated as you have done in the first sentence of Introduction.
2. In lines 55 the authors indicate that homoglutamate is a putative acyclic precursor of penicillins an cephalosporins. This word “putative” in this sentence is misleading; it is very well stablished in numerous articles that alpha aminoadipate (AAA) is a real precursor of penicillins and of cephalosporin C in *P. chrysogenum* and *C. acremonium,* respectively. Please clarify this point eliminating the word “putative” and providing more recent references. Also provide information on the enzymes producing AAA in actinobacteria and fungi.
3. In lines 69-70 it is indicated that there is no native pathway for the biosynthesis of alpha aminoadipate.This sentence is wrong; it is well known that there are pathways for AAA which are different in distinct microorganisms as actinobacteria and filamentous fungi. Please modify the sentence
4. In line 73 that authors state that AAA is an intermediate in the catabolism of lysine in mammals and in beta-lactam producing *actinomycetes* (Esmahan et al 1994). The work of Esmahan was made in filamentous fungi not in actinomycetes. This is an important microbiological mistake since actinomycetes are bacteria.
5. Lines 73-76. The authors say “However, this non proteinogenic amino can be…”. Please correct amino to aminoacid. The term “however” is not adequate since it means that there is a contradiction with the previous sentence, which is not the case. Change the term “however” to “in addition…”. Please correct also the reference of Luengo et al in line 76 and in the references list.
6. Lines 93-95 the ω-aminotransferase reaction which convert the alpha cetoadipic acid is into alpha amino adipic acid is probably the best approach to obtain unexpensive AAA- The mechanism of action of the ω aminotransferase is still poorly known but could be explored
7. In the second part of the introduction the author propos to use the phenylalanine dehydrogenase from *Thermoctinomyce*s to produce AAA from alpha cetoadipic. This is a surprising approach since the substrate of phenylalanine DH has a 9 carbons aromatic substrate that is deaminate to phenylpyruvate; this rection biochemically is quite different from that converting alpha-cetoadipate in AAA. The authors should clarify why they choose to use this reaction for the conversion but they do not approach the modification of enzymes related to the bacterial or fungal formation of AAA. The authors should give a clear study of the relative efficiency of AAA formation by the phenylalanine DH in relation to the natural enzymes forming AAA.
8. Line 118 and in the Results section the authors indicate that the affinity of the TiPDH for transamination of the 2-cetoadipate substrate is insufficient and therefore they try to improve the production by mutagenesis of two amino acids of the putative active center. This observation indicates that this is not the best starting point for the development of the high production of AAA.
9. In Materials and Methods the authors describe the quantification of AAA formation by HPLC but, as the authors indicate previously, homoglutamate (AAA) has not good UV absortion and the method utilized is not clear. In all classical studies amino acids are measured after derivatization with OPA or other UV emitting agent. Please indicate which is the method used in this article.
10. In the Discussion section the authors focus the discussion in the structure of the TiPDH and but they do not compare the active center of this enzyme with those of other alpha aminoadipic forming enzymes.
11. The authors in line 142 give the accession number of the nucleotides sequence of the TiPDH encoding gene, D00631. However, since in the article the authors constantly refer to the TiPDH protein the accession number of the protein should be easily accessible in the text. We found that the amino acids sequence shown in figure S2 does not corresponds to the sequences published by Ohshima et al or Tanaka et al. Is a different protein?. Please clarify.
12. In line 87 the authors refer to the work of Perez Llarena refering to an enzymatic reaction. This work is purely a description of gene sequences. The real work of purification and characterization of the enzyme in that of “de la Fuente et al., 1997”
