# Supplementary material for: Engineering of phenylalanine dehydrogenase from Thermoactinomyces intermedius for the production of a novel homoglutamate
Source: PLoS One. 2022 Mar 30;17(3):e0263784. doi: 10.1371/journal.pone.0263784 (PMC8967036; doi:10.1371/journal.pone.0263784)
Supplement: Attachment — Submitted filename: Response to Reviwers.docx [file pone.0263784.s007.docx]

Dear editor

We would like thanks to you and reviewers for their precious time to review our manuscript in deep and critically and for suggesting corrections that improved the quality of our manuscript. We are pleased to submit the revised manuscript entitled “**Engineering of Phenylalanine Dehydrogenase from *Thermoactinomyces Intermedius* for the Production of a novel Homoglutamate”** for consideration in **PLOS ONE**. On the following pages, you will find our response to the reviewer comments. The reviewer comments and suggestions are highlighted with red color and responses to each point with normal text. On behalf of my co-authors, I thank you for your consideration of this resubmission. We appreciate your time and look forward to your response.

Sincerely,

Dr. Muhammad Israr PhD, Postdoc (Corresponding Author)

Associate Professor, Department of Biology

The University of Haripur, KPK, Pakistan

**Reviewers comments and Responses**

**Reviewer**

**Comment 1:** In the abstract, the authors refer to the compound of interest as homoglutamate; however, this compound is usually named 2-aminoadipic acid or alpha aminoadipic acid. Please, use at least in the abstract the two names, so that the readers can identify the compound that is being investigated as you have done in the first sentence of the Introduction.

Response 1: We are grateful to the reviewer for taking his precious time to deeply review our manuscript which enhanced its quality.

Following the reviewer's suggestion, we have added two names of 2-aminoadipic acid in the Abstract section.

**Comment 2:**

Abstract: In line 55 the authors indicate that homoglutamate is a putative acyclic precursor of penicillins and cephalosporins. The word “putative” in this sentence is misleading; it is very well established in numerous articles that alpha aminoadipate (AAA) is a real precursor of penicillins and of cephalosporin C in *P. chrysogenum* and *C. acremonium,* respectively. Please clarify this point by eliminating the word “putative” and providing more recent references. Also, provide information on the enzymes producing AAA in actinobacteria and fungi.

Response 2: Thanks for this comment and suggestions. Yes, we are agreeing with the reviewer, the word “Putative” was replaced with “Well-known” as suggested by the reviewer and highlighted with red color in the track changes.

**Comment 3:**

In lines 69-70 it is indicated that there is no native pathway for the biosynthesis of alpha aminoadipate. This sentence is wrong; it is well known that there are pathways for AAA which are different in distinct microorganisms as actinobacteria and filamentous fungi. Please modify the sentence

Response 3: We are obliged to the reviewer for this comment and agree with his suggestion for correction. The sentence is modified and highlighted with red color in the track changes.

**Comment 4**:

In line 73 the authors state that AAA is an intermediate in the catabolism of lysine in mammals and beta-lactam producing *actinomycetes* (Esmahan et al 1994). The work of Esmahan was made in filamentous fungi, not in actinomycetes. This is an important microbiological mistake since actinomycetes are bacteria.

Response 4: We are grateful to the reviewer for pointing out this mistake. In the track changes, the sentence is corrected and the word “actinomycetes” is replaced with “Filamentous fungi” which is the work of Esmahan et al 1994.

**Comment 5:**

Lines 73-76. The authors say “However, this nonproteinogenic amino can be…”. Please correct amino to an amino acid. The term “however” is not adequate since it means that there is a contradiction with the previous sentence, which is not the case. Change the term “however” to “in addition…”. Please correct also the reference of Luengo et al in line 76 and the references list.

Response 5: We are grateful to the reviewer for this comment. The sentence and reference are modified accordingly and highlighted with red color in track changes.

**Comment 6**:

Lines 93-95 the ω-aminotransferase reaction which converts the alpha ketoadipic acid is into alpha-amino adipic acid is probably the best approach to obtain unexpansive AAA- The mechanism of action of the ω- aminotransferase is still poorly known but could be explored

Response 6: We are obliged to the reviewer for this comment. In some organisms (bacteria/fungi) the L-Lysine is converted into α-aminoadipic acid by two different pathways, in one pathway, lysine 2-ketoglutarate reductase results in the formation of saccharopine from lysine by extracting one amino group to be used as a nitrogen source. While in the second pathway the ω-aminotransferase converts D-lysine or L-lysine into 2-aminoadipic semialdehyde and 2-aminoadipic acid (homoglutamic acid). Both enzymes are prompted by L-lysine which is negatively regulated by NH_4_^+^ ions. The ω-aminotransferase from different species uses 2-ketoacids as amino group acceptors. The most common ones are 2-ketoadipate and 2-ketoglutarate resulting directly in the development of 2-aminoadipic acid and glutamic acid. We are agreed with the reviewer that ω--aminotransferase reaction which converts the alpha ketoadipic acid is into alpha-amino adipic acid is probably the best approach to obtain unexpansive AAA- The mechanism of action of the ω- aminotransferase is still poorly known but it could be explored.

**Comments 7:**

In the second part of the introduction, the author proposes to use the phenylalanine dehydrogenase from *Thermoactinomyc*es to produce AAA from alpha ketoadipic. This is a surprising approach since the substrate of phenylalanine DH has 9 carbons aromatic substrate that is delaminated to phenylpyruvate; this reaction biochemically is quite different from that converting alpha-ketoadipate in AAA. The authors should clarify why they choose to use this reaction for the conversion but they do not approach the modification of enzymes related to the bacterial or fungal formation of AAA. The authors should give a clear study of the relative efficiency of AAA formation by the phenylalanine DH in relation to the natural enzymes forming AAA.

Response 7: We are thankful to the reviewer for this comment. We have chosen reductive amination reaction strategies for the production of homoglutamate by proposing the phenylalanine dehydrogenase from *Thermoactinomyc*es using alpha ketoadipic which is an interesting route for the conversion of 2-ketoadipic acid catalyzed by NADH-dependent phenylalanine-dehydrogenase. The natural TiPDH enzyme affinity towards the transamination of 2-ketoadipic acid was previously found insufficient, therefore, we have chosen some amino acids sites in TiPDH and mutated which enhanced that catalytic activity for the production of AAA.

**Comment 8:**

Line 118 and in the Results section the authors indicate that the affinity of the TiPDH for transamination of the 2-cetoadipate substrate is insufficient and therefore they try to improve the production by mutagenesis of two amino acids of the putative active center. This observation indicates that this is not the best starting point for the development of the high production of AAA.

Response 8: We are obliged to the reviewer for this comment. In the introduction (line 111-116) and result section, we have modified the sentence in the track changes and highlighted it with red color according to the reviewer's suggestion.

**Comment 9:**

In Materials and Methods, the authors describe the quantification of AAA formation by HPLC but, as the authors indicate previously, homoglutamate (AAA) has not had good UV absorption and the method utilized is not clear. In all classical studies, amino acids are measured after derivatization with OPA or other UV emitting agents. Please indicate which is method used in this article.

Response 9: The authors are grateful to the reviewer for this comment. The method used in the article is explained in reference 36.

**Comment 10:**

In the Discussion section, the authors focus the discussion on the structure of the TiPDH and but they do not compare the active center of this enzyme with those of other alpha aminoadipic forming enzymes.

Response 10: We are obliged to the reviewer for raising this point. We have included some sentences of the revised manuscript (discussion section lines 454-456) which compared the active site domain of different alpha aminoadipic forming enzymes. We are the first to mutate the active site residues of TiPDH and no literature is available for comparing the active site of TiPDH with other alpha aminoadipic forming enzymes.

**Comment 11:**

The authors in line 142 give the accession number of the nucleotides sequence of the TiPDH encoding gene, D00631. However, since in the article, the authors constantly refer to the TiPDH protein the accession number of the protein should be easily accessible in the text. We found that the amino acids sequence shown in figure S2 does not correspond to the sequences published by Ohshima et al or Tanaka et al. Is it a different protein? Please clarify.

Response 11: We appreciate the reviewer for reviewing our article in deep. The Accession Number D00631 is for TiPDH which we used throughout the manuscript that can easily be assessable and can be recognized. The amino acid sequences in the S1 Figure are that of TiPDH. The protein sequences of TiPDH and those published by Oshima T and Takada H are the same proteins but from a different organism that has a resemblance in amino acid residues at the active site domain.

**Comment 12:**

In line 87 the authors refer to the work of Perez Llarena referring to an enzymatic reaction. This work is purely a description of gene sequences. The real work of purification and characterization of the enzyme in that of “de la Fuente et al., 1997”

Response 12: We are thankful to the reviewer for this comment. We are agreed with the reviewer that the reference of Perez Llarena is not for PDH enzymatic reaction, so in the revised manuscript we removed the Perez Llarena reference and replaced it with De la Fuente et al.,
