# Supplementary material for: Engineering of phenylalanine dehydrogenase from Thermoactinomyces intermedius for the production of a novel homoglutamate
Source: PLoS One. 2022 Mar 30;17(3):e0263784. doi: 10.1371/journal.pone.0263784 (PMC8967036; doi:10.1371/journal.pone.0263784)
Supplement: Attachment — Submitted filename: Response to Reviwers.docx [file pone.0263784.s008.docx]

Dear editor

We would like thanks to you and reviewers for their precious time to review our manuscript in deep and critically and for suggesting corrections that improved the quality of our manuscript. We are pleased to submit the revised manuscript entitled “**Engineering of Phenylalanine Dehydrogenase from *Thermoactinomyces Intermedius* for the Production of a novel Homoglutamate”** for consideration in **PLOS ONE**. On the following pages, you will find our response to the reviewer comments. The reviewer comments and suggestions are highlighted with red color and responses to each point with normal text. On behalf of my co-authors, I thank you for your consideration of this resubmission. We appreciate your time and look forward to your response.

Sincerely,

Dr. Muhammad Israr PhD, Postdoc (Corresponding Author)

Associate Professor, Department of Biology

The University of Haripur, KPK, Pakistan

**Reviewers comments and Responses**

**Reviewer**

**Comment 1:** In Fig. 1 a and 1 b, km and Vmax units should be added.

Response 1: We are grateful to the reviewer for pointing out this mistake. The units of Km and Vmax are added in the revised manuscript.

**Comment 2:**

For the effect of sucrose and trehalose, previous results on other enzymes are stronger, please discuss: International journal of biological macromolecules 43 (2), 187-191, 2008.

Response 2: Thanks for this comment. In line 404-406 of result part, we have added a sentence with reference for comparing the effect of osmolytes such as sucrose on the thermal stability of previously reported enzyme (firefly luciferase) with our results.

**Comment 3:**

Other variants of phenylalanine dehydrogenase have been reported and should be used

In introduction part: Archives of biochemistry and biophysics 635, 44-51, 2017

Response 3: We are obliged to the reviewer for this comment. In line 97-98 of introduction part, a reference for reporting phenylalanine dehydrogenase from *Bacillus badius* has added accordingly.
